# Impact of Unsaturated Fatty Acids on Cytokine-Driven Endothelial Cell Dysfunction

**DOI:** 10.3390/ijms18122739

**Published:** 2017-12-16

**Authors:** Simon Trommer, Anja Leimert, Michael Bucher, Julia Schumann

**Affiliations:** Clinic for Anesthesiology and Surgical Intensive Care, University Hospital Halle (Saale), 06120 Halle (Saale), Germany; simon.trommer@student.uni-halle.de (S.T.); anja.leimert@uk-halle.de (A.L.); michael.bucher@uk-halle.de (M.B.)

**Keywords:** endothelial dysfunction, PUFA, cytokines, adhesion molecules, coagulation factors

## Abstract

Polyunsaturated fatty acids (PUFA) are reported to exert prophylactic and acute therapeutic effects in diseases linked to endothelial dysfunction. In the present study, the consequences of a PUFA enrichment of endothelial cells (cell line TIME) on cell viability, expression of the cytokines interleukin-6 (IL-6), interleukin-8 (IL-8), granulocyte-macrophage colony-stimulating factor (GM-CSF), and monocyte chemoattractant protein 1 (MCP-1), synthesis of the adhesion molecules intercellular adhesion molecule 1 (ICAM-1) and vascular adhesion molecule 1 (VCAM-1), and production of the coagulation factors plasminogen activator inhibitor-1 (PAI-1), von Willebrand factor (vWF), and tissue factor (TF) was analyzed in parallel. PUFA of both the n3 and the n6 family were investigated in a physiologically relevant concentration of 15 µM, and experiments were performed in both the presence and the absence of the pro-inflammatory cytokines interleukin-1β (IL-1β), tumor necrosis factor-α (TNF-α), and interferon-γ (IFN-γ). Supplementation of the culture medium with particular fatty acids was found to have a promoting effect on cellular production of the cytokines IL-6, IL-8, GM-CSF, and MCP-1. Further on, PUFA treatment in the absence of a stimulant diminished the percentage of endothelial cells positive for ICAM-1, and adversely affected the stimulation-induced upregulation of VCAM-1. Cell viability and production of coagulation factors were not or only marginally affected by supplemented fatty acids. Altogether, the data indicate that PUFA of either family are only partially able to counterbalance the destructive consequences of an endothelial dysfunction.

## 1. Introduction

The endothelium represents a single cell layer lining all vessels. It forms an interface between blood and tissues, and is an important barrier for the passage of molecules or cells to the underlying interstitium. Endothelial cells are involved in the regulation of various physiological processes. Among other functions, the endothelium mediates vasomotor tone, maintains blood fluidity, and regulates cellular and nutrient trafficking. Due to their strategic localization, endothelial cells are highly active, constantly sensing alterations in their local environment. The vast endothelial surface provides ample sites for interactions with other cells, both blood-borne or from the vessel wall. In health, the endothelium represents a non-adhesive, anti-coagulant, and non-thrombogenic surface. On exposure to environmental stimuli such as inflammatory mediators, however, endothelial cells undergo profound changes in gene expression, thereby rapidly modulating structure and function. Upon activation, endothelial cells synthesize and release pro-inflammatory cytokines (e.g., tumor necrosis factor-α (TNF-α), granulocyte-macrophage colony-stimulating factor (GM-CSF), monocyte chemoattractant protein 1 (MCP-1), and the interleukins IL-1β, IL-6, and IL-8), produce reactive oxygen species and nitrogen intermediates, and promote leukocyte adhesion and extravasation via increased expression of intercellular adhesion molecule 1 (ICAM-1) and vascular adhesion molecule 1 (VCAM-1) [1,2,3,4,5,6]. Further functional alterations include programmed cell death, loss of barrier function, and a shift in hemostatic balance in favor of a pro-coagulant state mediated by an enhanced synthesis of plasminogen activator inhibitor-1 (PAI-1), von Willebrand factor (vWF), and tissue factor (TF) [2,3]. Endothelial activation occurs as a normal adaptive response, allowing the cells to participate in various inflammatory reactions, and to contribute to the containment and elimination of invading pathogens. Misdirected activation, however, results in endothelial dysfunction that is linked to organ damage and critical injury. In sepsis, acute and systemic endothelial dysfunction is a major problem contributing to microvascular leakage and multiple organ failure [2,4,7,8]. In addition, dysfunctional endothelial cells, which are continuously activated by local chronic inflammatory processes, are considered as a starting point in the development of atherosclerotic lesions [9,10,11].

Enrichment of diets with specific lipid compositions has long attracted interest for modulating inflammatory conditions. Dietary polyunsaturated fatty acids (PUFA) serve as precursors for the inflammation-mediating eicosanoids and resolvins, and represent direct ligands of transcription factors associated to immune response [12,13,14]. In addition, supplemented PUFA are known to be incorporated into plasma membrane phospholipids at these modulating cellular signaling events [12]. For instance, activity of the transcription factor nuclear factor kappa-light-chain-enhancer of activated B cells (NFκB), which plays a key role in regulating inflammatory reactions, has been shown to be impaired by unsaturated fatty acids [15]. In line with that, PUFA seem to be of clinical relevance, yet the family of a fatty acid (n3 vs. n6) appears to be of special importance. Infusion of long-chain n3 PUFA (in contrast to n6 PUFA) in parenteral nutrition given to either laboratory animals or patients suffering from sepsis is reported to result in genuine clinical benefits [16,17]. Dietary supply of n3 PUFA is also discussed as beneficial treatment of atherosclerosis development [11,18,19,20].

Despite the reported prophylactic and acute therapeutic effects of n3 PUFA in diseases linked to endothelial dysfunction, comprehensive data concerning the impact of unsaturated fatty acids on endothelial cell functionality are missing. Concerning the effects of n3 PUFA on endothelial expression of adhesion molecules, the reported data hint toward an inhibitory action of the fatty acids on cytokine-induced upregulation of ICAM-1 and VCAM-1 that might take place on the transcriptional level mediated by NFκB [18,19,20,21,22,23]. Further on, there are indications that n3 PUFA decrease endothelial formation of reactive oxygen species [20,23]. Other characteristic features of endothelial dysfunction such as imbalanced expression of coagulation factors, elevated cytokine production as well as endothelial cell damage and the possibility to influence them by PUFA supplementation still need to be elucidated. The present study was undertaken using a human telomerase immortalized endothelial cell line (TIME), which fulfills endothelial characteristics with respect to the expression of cytokines, adhesion molecules, and coagulation factors, to fill this gap of knowledge comparing several PUFA not only of the n3 but also of the n6 family.

## 2. Results

### 2.1. Fatty Acid Composition

Culture of the human endothelial cell line TIME in medium containing 15 µmol/L linolenic acid (LNA, C18:3n3), eicosapentaenoic acid (EPA, C20:5n3), and docosahexaenoic acid (DHA, C22:6n3), respectively, resulted in a significant enrichment in the content of cellular n3 PUFA (C18:3n3, C20:3n3, C20:4n3, C20:5n3, C22:4n3, C22:5n3, C22:6n3), while culture in medium containing 15 µmol/L linoleic acid (LA, C18:2n6) or arachidonic acid (AA, C20:4n6) resulted in a significant enrichment in the content of n6 PUFA (C18:2n6, C18:3n6, C20:3n6, C20:4n6, C22:3n6, C22:4n6, C22:5n6) (Table 1). This is attributable to a considerable rise in the content of the respective supplemented fatty acid as well as of its derivatives due to chain elongation and desaturation reactions (Table 1). Of note, the proportions of fatty acids belonging to the saturated, n7, and n9 fatty acid families were altered as well. The content of saturated fatty acids increased and the content of monounsaturated fatty acids of the n7 and the n9 family decreased. Overall, these changes in cellular fatty acid composition resulted in an enhancement in calculated methylene bridge index (MBI), which is an indicator of unsaturation (Table 1). It is to emphasize that, with the exception of cells cultured in the presence of AA, total fatty acid content was not significantly changed by PUFA supplementation (Table 1).

### 2.2. Cell Viability

**Figure 1:**
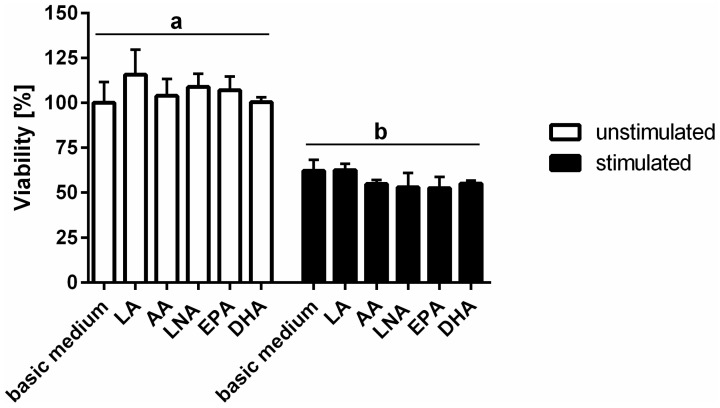
TIME viability subject to stimulation and polyunsaturated fatty acids (PUFA) supplementation. Cellular viability was analyzed luminometrically using the RealTime-Glo MT Cell Viability Assay from Promega (Mannheim, Germany). TIME were cultured for 144 h in basic medium as well as in medium supplemented with 15 µmol/L α-linolenic acid (LNA, C18:3n3), eicosapentaenoic acid (EPA, C20:5n3), docosahexaenoic acid (DHA, C22:6n3), linoleic acid (LA, C18:2n6), and arachidonic acid (AA, C20:4n6) respectively. Stimulation was performed in the last 24 h of fatty acid supplementation by addition of the cytokines interleukin-1β (IL-1β), tumor necrosis factor-α (TNF-α), and interferon-γ (IFN-γ) (5 ng/mL each). Data are mean ± S.D. (*N* = 6, *n* = 1). Superscript letters across a row denote significant differences.

Treatment of TIME using a cytokine mixture comprising IL-1β, TNF-α, and IFN-γ brought about a significant reduction in cell viability (Figure 1). PUFA enrichment of TIME, however, was found not to interfere with cell viability. There were no differences in the percentage of viable cells cultured either in basic medium or in medium supplemented with the fatty acids LNA, EPA, DHA, LA, and AA, respectively, regardless the cellular activation status (Figure 1). The cytokine-mediated drop in cell viability remained unaffected by PUFA supplementation (Figure 1).

### 2.3. Cytokines

**Figure 2:**
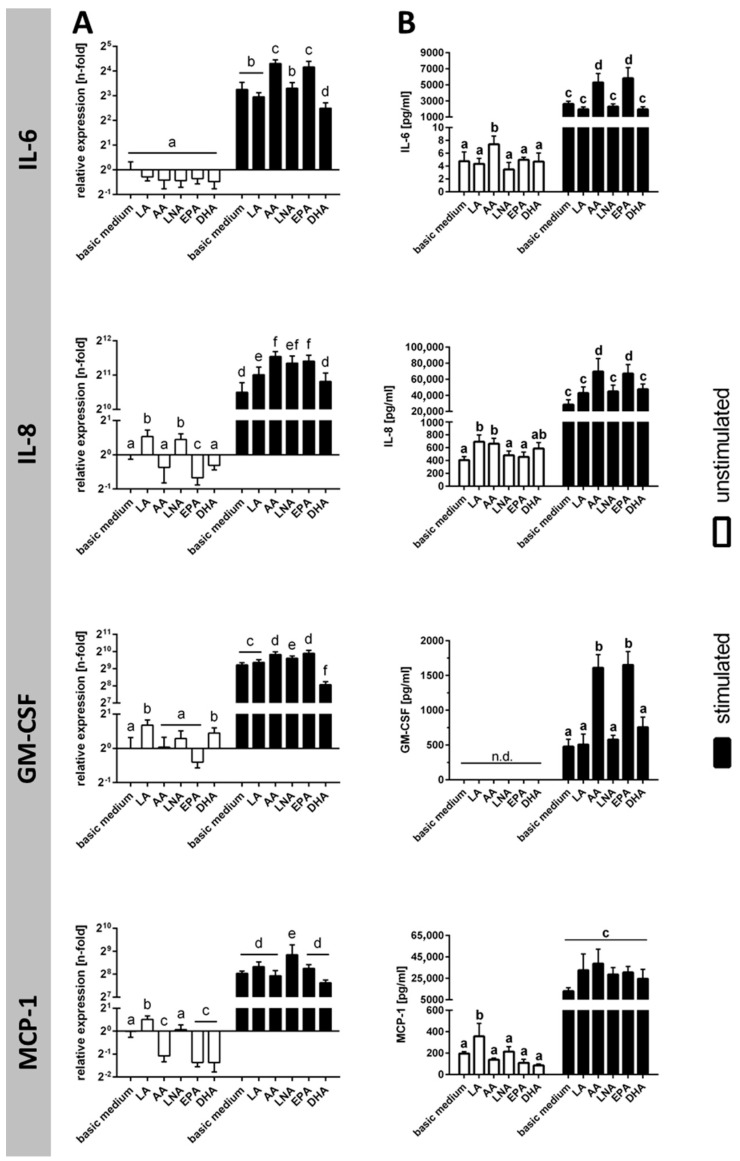
Cytokine synthesis subject to stimulation and PUFA supplementation. Cytokine production was analyzed (**A**) on mRNA level by quantitative real-time PCR (*N* = 6, *n* = 3) and (**B**) on protein level by ELISA (*N* = 6, *n* = 2). TIME were cultured for 144 h in basic medium as well as in medium supplemented with 15 µmol/L α-linolenic acid (LNA, C18:3n3), eicosapentaenoic acid (EPA, C20:5n3), docosahexaenoic acid (DHA, C22:6n3), linoleic acid (LA, C18:2n6), and arachidonic acid (AA, C20:4n6), respectively. Stimulation was performed in the last 24 h of fatty acid supplementation by addition of the cytokines IL-1β, TNF-α, and IFN-γ (5 ng/mL each). Data are mean ± S.D. Superscript letters across a row denote significant differences.

Stimulation of TIME was associated with a marked rise in the expression of the cytokines IL-6, IL-8, GM-CSF, and MCP-1, which was evident both on RNA level (Figure 2A) and on protein level (Figure 2B). In cell culture supernatants, IL-6 content increased 552 times, IL-8 content increased 70 times, and MCP-1 content increased 66 times due to endothelial cell activation (Figure 2B). Concentrations of GM-CSF protein were below detection limit for unstimulated TIME and reached a value of almost 500 pg/mL upon stimulation (Figure 2).

Supplementation of the culture medium with particular fatty acids was found to have a promoting effect on cellular cytokine production (Figure 2A,B). Enrichment of unstimulated TIME with the PUFA LA and AA resulted in an increase of IL-6, IL-8, and MCP-1 in cell culture supernatants by factors of 1.6 to 1.8 (Figure 2B). In addition, the PUFA EPA and AA further enhanced the stimulation-induced production of IL-6, IL-8, and GM-CSF by factors of 2.0 to 3.5 (Figure 2B). Two of the investigated fatty acids (LNA and DHA), however, did not have an impact on cytokine synthesis rates regardless of cellular activation status at least on the protein level (Figure 2A,B).

### 2.4. Adhesion Molecules

**Figure 3:**
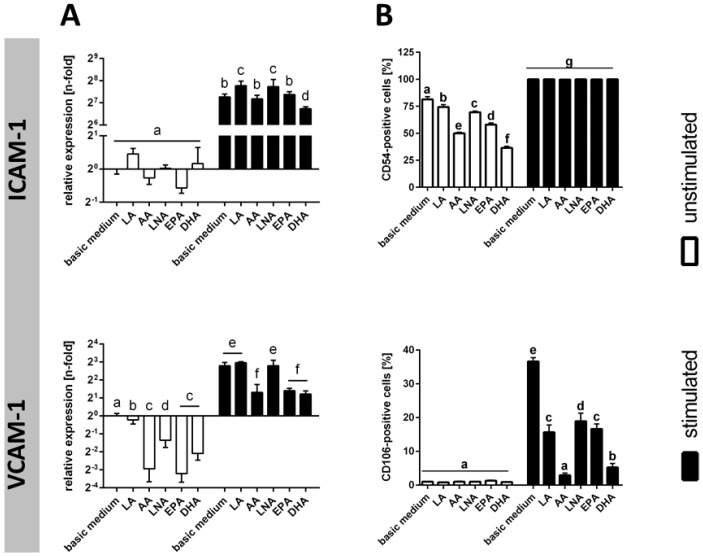
Expression of adhesion molecules subject to stimulation and PUFA supplementation. Expression of the adhesion molecules ICAM-1 (CD54) and VCAM-1 (CD106) was analyzed (**A**) on mRNA level by quantitative real-time PCR (*N* = 6, *n* = 3) and (**B**) on protein level by flow cytometry (*N* = 6, *n* = 2). TIME were cultured for 144 h in basic medium as well as in medium supplemented with 15 µmol/L alpha-linolenic acid (LNA, C18:3n3), eicosapentaenoic acid (EPA, C20:5n3), docosahexaenoic acid (DHA, C22:6n3), linoleic acid (LA, C18:2n6), and arachidonic acid (AA, C20:4n6), respectively. Stimulation was performed in the last 24 h of fatty acid supplementation by addition of the cytokines IL-1β, TNF-α, and IFN-γ (5 ng/mL each). Data are mean ± S.D. Superscript letters across a row denote significant differences.

Cytokine-mediated activation of TIME resulted in a significant up-regulation of ICAM-1 and VCAM-1 mRNA levels (Figure 3A). This was accompanied by an augmentation of the number of cells displaying ICAM-1 and VCAM-1 on their surface (Figure 3B). Due to stimulation, the percentage of ICAM-1-positive cells increased from 81% to 100%, and the percentage of VCAM-1-positive cells increased from 1% to 37% (Figure 3B).

The consequences of a PUFA supplementation differed depending on cellular activation status and the type of adhesion molecule. In the absence of a stimulant, the percentages of cells positive for ICAM-1 were significantly diminished by additional PUFA supply with DHA (36%) and AA (50%) having the biggest effect (Figure 3B). Upon stimulation, however, no moderating effect of PUFA on cellular ICAM-1 expression was seen (Figure 3B). VCAM-1 was expressed in unstimulated cells at a very low level, which was not influenced by additional PUFA supply (Figure 3B). In contrast, the stimulation-induced up-regulation of VCAM-1 was adversely affected by cellular PUFA enrichment. The PUFA DHA (5%) and AA (3%) emerged to be particularly effective (Figure 3B).

### 2.5. Coagulation Factors

**Figure 4:**
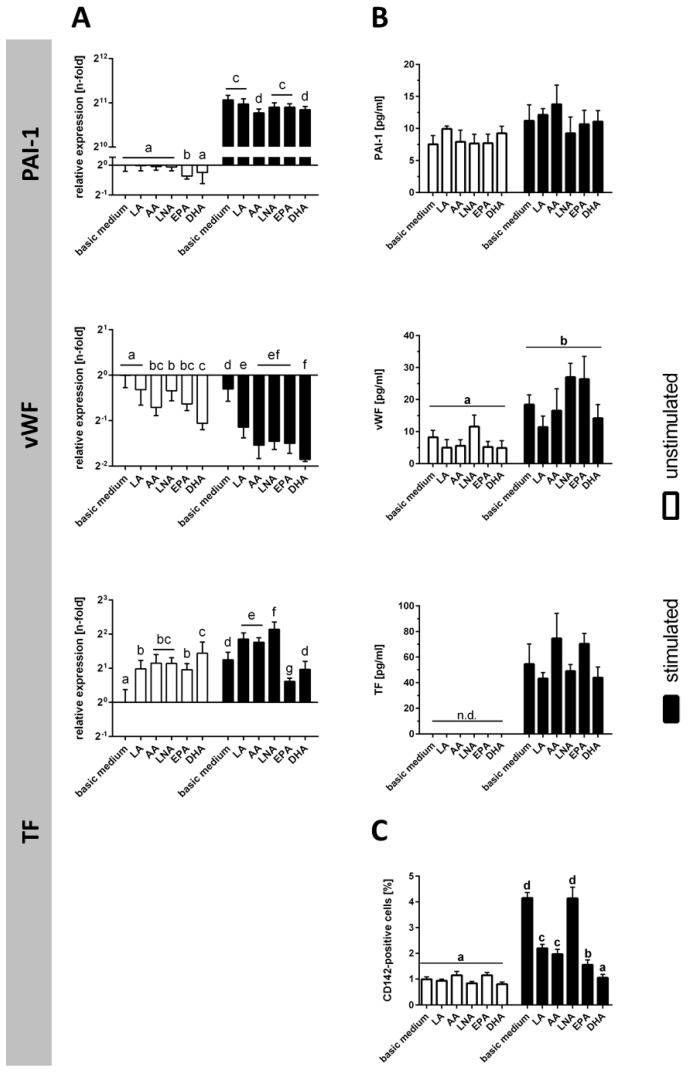
Expression of coagulation factors subject to stimulation and PUFA supplementation. Expression of the coagulation factors PAI-1, vWF, and TF (both soluble and membrane-bound) was analyzed (**A**) on mRNA level by quantitative real-time PCR (*N* = 6, *n* = 3) and on protein level (*N* = 6, *n* = 2) by (**B**) ELISA (PAI-1, vWF, soluble TF) and (**C**) flow cytometry (membrane-bound TF). TIME were cultured for 144 h in basic medium as well as in medium supplemented with 15 µmol/L α-linolenic acid (LNA, C18:3n3), eicosapentaenoic acid (EPA, C20:5n3), docosahexaenoic acid (DHA, C22:6n3), linoleic acid (LA, C18:2n6), and arachidonic acid (AA, C20:4n6), respectively. Stimulation was performed in the last 24 h of fatty acid supplementation by addition of the cytokines IL-1β, TNF-α, and IFN-γ (5 ng/mL each). Data are mean ± S.D. Superscript letters across a row denote significant differences.

Cytokine treatment of TIME resulted in the mRNA up-regulation of PAI-1 and TF, which was contrasted by an mRNA down-regulation of vWF (Figure 4A). In cell culture supernatants, there was a moderate rise in PAI-1 content, and a clear augmentation in the content of vWF and soluble TF (Figure 4B). In addition, there was a fourfold increase in the percentage of cells positive for membrane-bound TF, which nevertheless remained in the minority (Figure 4C).

PUFA enrichment of cells did not affect protein expression levels of PAI-1, vWF and soluble TF (despite some impact on mRNA expression levels) (Figure 4A,B). The stimulation-induced up-regulation of TF-expressing cells, however, was abolished by fatty acid supplementation (with exception of LNA) (Figure 4C).

## 3. Discussion

Accumulating evidence suggests that cytokines play an important role in endothelial dysfunction. Endothelial cells are both a target and a source of cytokines. In an autocrine manner, cytokine-mediated endothelial activation is reported to trigger the secretion of additional inflammatory mediators into the circulation [1,3,5]. Consequently, we observed significantly increased concentrations of IL-6, IL-8, GM-CSF, and MCP-1 in cell culture supernatants following exposure of endothelial cells to the pro-inflammatory cytokines IL-1β, TNF-α, and IFN-γ. Furthermore, cytokines are described to modulate endothelial hemostatic properties as well as the expression of adhesion molecules [1,2,3,6]. In line with that in our experimental setting, the coagulation factors vWF and TF were clearly enhanced in the context of cytokine stimulation. The number of endothelial cells displaying ICAM-1 and VCAM-1 rose as well. The upregulated expression of adhesion molecules in tandem with a sluggish blood flow is known to enhance endothelium–leukocyte interaction and to facilitate rolling, adherence, and transmigration of immune cells into the underlying tissue [2,24]. Endothelial cell activation is not necessarily linked to disease. Nevertheless, exaggerated vascular permeability may result in capillary leakage and organ failure. A factor contributing to this is the cytokine-mediated apoptosis of endothelial cells [2]. In fact, in our experiments, the number of viable endothelial cells was almost halved in the presence of the pro-inflammatory cytokines IL-1β, TNF-α, and IFN-γ.

Membrane phospholipid composition is subject to external fatty acid supply [12,17,25]. For this reason, blood-borne cells as well as cells with constant blood contact such as endothelial cells are directly influenced by dietary PUFA intake. In our experimental protocol, we successfully simulated these natural conditions. Supplementation of endothelial cells with LNA, EPA, DHA, LA or AA resulted in enrichment with unsaturated fatty acids. Interestingly, cellular incorporation of an added fatty acid was accompanied by an increase in the content of its desaturation and elongation derivatives. We want to emphasize that the PUFA concentration used in our experiments (15 µM) is of physiological relevance. It is well in the range of these fatty acids in the context of oral supplementation using fish oil capsules [25]. Under conditions of parenteral nutrition, even higher PUFA blood content will be achieved [25].

Our investigations provide evidence that cytokine-driven endothelial dysfunction at least in part is modulated by unsaturated fatty acids. Distinct effects were seen on endothelial cytokine release as well as the expression of adhesion molecules. Firstly, a promoting effect of EPA and AA on the stimulation-induced endothelial production of the cytokines IL-6, IL-8, and GM-CSF was found. However, the potency of the fatty acids on endothelial cytokine release was small in relation to the stimulating effect mediated by IL-1β, TNF-α, and IFN-γ. Secondly, the stimulation-mediated expression of VCAM-1 was hampered by all PUFA tested. So far, suppression of the cytokine-induced upregulation of VCAM-1 by endothelial cells has been solely shown for the n3 PUFA DHA and EPA [18,19,20,21,22]; our data first provide evidence that this effect is also true for members of the n6 family. It should be noted that, due to cytokine stimulation, the endothelial cells unanimously displayed ICAM-1 on their surface. PUFA enrichment of cells did not influence on this, which might be related to the low, yet physiologically relevant, concentration used in our experiments. There were no indications of a modulating action of the fatty acids on cytokine-induced apoptosis or release of soluble coagulation factors (PAI-1, vWF, TF). Stimulation-mediated upregulation of membrane-bound TF, however, was prevented by PUFA enrichment of endothelial cells. Taken together, our data indicate that PUFA of either family are only partially able to counterbalance the destructive consequences of an endothelial dysfunction. The comprehensive approach in terms of testing many different PUFA further shows that fatty acids of both the n3 and the n6 family mediate similar effects on endothelial reactions.

## 4. Materials and Methods

### 4.1. Materials

All chemicals and reagents were obtained from Sigma-Aldrich (Taufkirchen, Germany) unless noted otherwise. Cell culture flasks and plates were purchased from Greiner Bio-One (Frickenhausen, Germany). Microvascular endothelial growth medium as per customer specification according to the American Type Culture Collection (ATCC) recommendations was acquired from Provitro AG (Berlin, Germany).

### 4.2. Cell Culture, Fatty Acid Supplementation, and Stimulation

The human telomerase-immortalized microvascular endothelial cell line TIME (ATCC number: CRL-4025) was used. TIME were cultured in basal microvascular endothelial growth medium enriched with 5 ng/mL VEGF, 5 ng/mL EGF, 5 ng/mL FGF, 15 ng/mL IGF-1, 10 mM l-glutamine, 0.75 U/mL heparin sulfate, 1 µg/mL hydrocortisone hemisuccinate, 50 µg/mL ascorbic acid, 5% *v*/*v* FCS, 12.5 µg/mL blasticidin, and 0.1% *v*/*v* ethanol (basic medium). The fatty acids LNA (C18:3n3), EPA (C20:5n3), DHA (C22:6n3), LA (C18:2n6) or AA (C20:4n6) (all Biotrend, Köln, Germany) were included in the culture medium in concentrations of 15 µmol/L using ethanol as a vehicle (0.1% *v*/*v* final ethanol concentration). Cells were cultured in the enriched media in 75 cm^2^ cell culture flasks for 144 h at 37 °C and 5% CO_2_ in a humidified atmosphere. Periods of supplementation were proven to result in a membrane fatty acid steady state. Stimulation of cells was performed in the last 24 h of fatty acid supplementation by addition of the cytokines IL-1β, TNF-α, and IFN-γ (all PeproTech, Hamburg, Germany) each in a concentration of 5 ng/mL.

### 4.3. Gas Chromatography

Fatty acid composition was analyzed by lipid extraction and subsequently gas chromatography in cooperation with the Institute of Biochemistry, Faculty of Veterinary Medicine, University of Leipzig. The membrane lipids were trans-esterified with 500 µL methanolic HCl, 250 µL *n*-hexane, and 500 µL internal standard (0.8 mg di-C17-phosphatidylcholine in 1 mL methanol with 0.2% butylhydroxyltoluol as antioxidant). After cooling-off, 500 µL *n*-hexane and 1 mL aqua dest. were added. The upper hexane phase was evaporated with nitrogen. The fatty acid methylesters (FAME) were taken up in 60 µL *n*-hexane. An aliquot of 1 µL was injected on-column on a Varian CP 3800 gas chromatograph (Varian, Darmstadt, Germany) equipped with an Omegawax TM 320 column (0.32 mm internal diameter, 30 m length) (Sulpeco, Bellefonte, PA, USA). The column temperature was 200 °C. Measurements are representatives of six independent experiments for each fatty acid supplementation. Based on the ratio of a fatty acid (weight %) detected by gas chromatography and the number of its bis-allyl-methylene positions the Methylene Bridge Index (MBI) was calculated [26].

### 4.4. Viability Assay

TIME were cultured, supplemented, and stimulated as described above. Cell viability was determined using the RealTime-Glo MT Cell Viability Assay (Promega, Mannheim, Germany) according to the instructions of the manufacturer. Luminescence was read on an Infinite M200 plate reader and analysis done using the i-control^TM^ software (all Tecan Group, Männedorf, Switzerland). Measurements are representatives of six independent experiments for each fatty acid supplementation of unstimulated and stimulated cells.

### 4.5. Quantitative Real-Time PCR

TIME were cultured, supplemented, and stimulated as described above. Gene expression was analyzed by a SYBR Green-based quantitative real-time PCR. Total RNA was extracted using the InviTrap Spin Universal RNA Mini Kit (Stratec Biomedical, Birkenfeld, Germany). Complementary DNA was synthesized using the qScript cDNA SuperMix, and quantitative real-time PCR was performed using the PerfeCTa SYBR Green FastMix (both Quanta BioSciences, Gaithersburg, MD, USA). Genes of interest amplified are IL-6, IL-8, GM-CSF, MCP-1, PAI-1, vWF, TF, ICAM-1, and VCAM-1; reference genes amplified are β-actin and glyceraldehyde phosphate dehydrogenase (GAPDH). Primer sequences and thermal cycling conditions used can be found in Table 2. Positive controls (i.e., human heart aorta total RNA, Takara Bio Europe SAS, Saint-Germain-en-Laye, France) as well as negative controls (i.e., no template control, no reverse transcriptase control) were performed for each run. Thermal cycling was carried out on the 7900HT Fast Real-Time PCR System (Thermo Fisher Scientific, Dreieich, Germany). To take account of individual amplification efficiencies for every PCR, data analysis was conducted based on the gene expression’s CT difference (GED) method using an efficiency adjusted normalization factor calculated as the geometric mean of the two reference genes β-actin and GAPDH [27]. Calculation of linear regression was carried out by means of the publicly available program LinRegPCR [28]. Measurements were performed in triplicates and are representatives of six independent experiments for each fatty acid supplementation of unstimulated and stimulated cells.

### 4.6. ELISA

TIME were cultured, supplemented, and stimulated as described above. GM-CSF, IL-6 (Sigma-Aldrich Taufkirchen, Germany), IL-8, MCP-1, PAI-1, vWF, and soluble TF (R&D Systems, Wiesbaden, Germany) were detected in supernatants using suitable human ELISA kits according to the instructions of the manufacturer. Absorbance was read on an Infinite M200 plate reader at 450 nm and analysis done using the i-control^TM^ software, version 2.11 (all Tecan Group, Männedorf, Switzerland). Measurements were performed in duplicates and are representatives of six independent experiments for each fatty acid supplementation of unstimulated and stimulated cells.

### 4.7. Flow Cytometry

TIME were cultured, supplemented, and stimulated as described above. Cells were stained for the surface marker anti-CD54-APC (= ICAM-1), anti-CD106-PE (= VCAM-1) (BD Biosciences, Heidelberg, Germany), and anti-CD142-FITC (= membrane-bound TF) (Miltenyi Biotech, Bergisch Gladbach, Germany) and fixed in 1% formaldehyde in PBS. In all cases, an Fc block (Miltenyi Biotech, Bergisch Gladbach, Germany) was used. Specificity of staining was verified via isotope control carried along for the antibodies used. Cells were analyzed on a FACSCalibur using Cellquest Pro software (version, all Becton Dickinson, Heidelberg, Germany). Measurements were performed in duplicate and are representatives of six independent experiments for each fatty acid supplementation of unstimulated and stimulated cells.

### 4.8. Statistical Analysis

Data are shown as means ± standard deviation (S.D.). One-way analysis of variance followed by Holm–Sidak corrected multiple comparison was used to identify significant differences between means. The statistical analysis was carried out by means of the program GraphPad Prism 6 (GaphPad Software, La Jolla, CA, USA). In all cases, *p* < 0.05 was assumed to indicate significant differences.

## Figures and Tables

**Table 1:** Fatty acid patterns (nmol/L × 10^7^ cells) and methylene bridge index (MBI) of TIME cultured for 144 h in basic medium as well as in medium supplemented with 15 µmol/L alpha-linolenic acid (LNA, C18:3n3), eicosapentaenoic acid (EPA, C20:5n3), docosahexaenoic acid (DHA, C22:6n3), linoleic acid (LA, C18:2n6), and arachidonic acid (AA, C20:4n6), respectively, as determined by gas chromatography. Data are mean ± S.D. (*N* = 6, *n* = 1).

Fatty Acid	Basic Medium	LNA	EPA	DHA	LA	AA
C18:3n3	n.d.	108.2 ± 9.7 ^b^	0.9 ± 0.1 ^a^	0.4 ± 0.1 ^a^	n.d.	0.9 ± 0.2 ^a^
C20:3n3	0.2 ± 0.2 ^a^	37.2 ± 4.9 ^b^	0.6 ± 0.1 ^a^	0.5 ± 0.1 ^a^	0.3 ± 0.1 ^a^	0.8 ± 0.2 ^a^
C20:4n3	1.2 ± 0.2 ^a^	49.6 ± 4.2 ^c^	15.1 ± 0.7 ^b^	2.5 ± 0.2 ^a^	0.4 ± 0.1 ^a^	1.7 ± 0.5 ^a^
C20:5n3	8.9 ± 0.5 ^a^	80.9 ± 8.2 ^c^	116.0 ± 3.8 ^d^	33.8 ± 3.4 ^b^	1.5 ± 0.1 ^a^	3.2 ± 0.6 ^a^
C22:4n3	n.d.	7.5 ± 0.8 ^c^	1.8 ± 0.1 ^b^	1.0 ± 0.2 ^a^	n.d.	n.d.
C22:5n3	37.1 ± 2.6 ^a^	134.5 ± 14.5 ^b^	242.3 ± 10.0 ^c^	40.7 ± 5.9 ^a^	28.3 ± 3.3 ^a^	35.5 ± 3.6 ^a^
C22:6n3	42.4 ± 2.9 ^a^	42.2 ± 4.6 ^a^	62.5 ± 2.5 ^b^	129.7 ± 15.4 ^c^	30.2 ± 3.6 ^a^	23.4 ± 5.2 ^a^
C18:2n6	10.6 ± 0.8 ^a^	16.3 ± 1.5 ^a^	16.1 ± 0.6 ^a^	15.2 ± 1.8 ^a^	139.4 ± 17.3 ^b^	20.9 ± 4.0 ^a^
C18:3n6	1.2 ± 0.2 ^a^	1.7 ± 0.2 ^a^	2.0 ± 0.2 ^a^	1.4 ± 0.1 ^a^	5.3 ± 0.7 ^b^	14.3 ± 1.6 ^c^
C20:3n6	13.7 ± 1.3 ^a^	10.2 ± 1.1 ^a^	10.8 ± 0.3 ^a^	16.7 ± 1.8 ^a^	40.0 ± 4.9 ^b^	64.4 ± 6.3 ^b^
C20:4n6	51.2 ± 4.0 ^a^	31.6 ± 3.0 ^a^	31.4 ± 1.9 ^a^	44.6 ± 5.5 ^a^	97.9 ± 12.3 ^a^	368.8 ± 85.3 ^b^
C22:3n6	n.d.	n.d.	n.d.	n.d.	6.1 ± 1.0 ^a^	6.8 ± 2.4 ^a^
C22:4n6	9.0 ± 0.6 ^a^	5.3 ± 0.5 ^a^	6.3 ± 0.3 ^a^	5.1 ± 0.9 ^a^	58.8 ± 7.0 ^b^	303.0 ± 60.4 ^c^
C22:5n6	3.0 ± 0.2 ^a^	1.4 ± 0.3 ^a^	1.9 ± 0.2 ^a^	2.3 ± 0.3 ^a^	25.4 ± 3.1 ^b^	35.5 ± 11.3 ^c^
Total fatty acids	967 ± 75 ^a^	1146 ± 105 ^a^	1234 ± 45 ^a^	1105 ± 138 ^a^	1118 ± 137 ^a^	1681 ± 214 ^b^
Total saturated	380 ± 28 ^a^	423 ± 37 ^a^	507 ± 19 ^b^	470 ± 59 ^b^	466 ± 58 ^b^	552 ± 56 ^b^
Total n3	90 ± 6 ^a^	467 ± 45 ^c^	442 ± 16 ^c^	210 ± 25 ^b^	61 ± 7 ^a^	67 ± 9 ^a^
Total n6	87 ± 6 ^a^	66 ± 6 ^a^	69 ± 2 ^a^	85 ± 10 ^a^	365 ± 41 ^b^	779 ± 164 ^c^
Total n7	91 ± 8 ^a^	50 ± 5 ^b^	56 ± 2 ^b^	82 ± 10 ^a^	52 ± 6 ^b^	64 ± 7 ^b^
Total n9	293 ± 26 ^a^	124 ± 12 ^c^	144 ± 5 ^c^	237 ± 30 ^b^	134 ± 16 ^c^	163 ± 16 ^c^
MBI	76 ± 2 ^a^	152 ± 2 ^d^	163 ± 1 ^e^	111 ± 2 ^c^	102 ± 1 ^b^	160 ± 6 ^e^

Superscript letters across a row denote significant differences. n.d. = below detection limit.

**Table 2:** Target, primer sequence, product size, annealing temperature (X), and elongation time (Y) used for quantitative real-time PCR.

Target	Primer Sequence (5′→3′)	Product Size (bp)	Annealing Temperature (°C)	Extension Time (s)
IL-6	AAGCCAGAGCTGTGCAGATG CTGGCATTTGTGGTTGGGTC	106	60	10
IL-8	CCTGATTTCTGCAGCTCTGTG CCAGACAGAGCTCTCTTCCAT	197	56	20
GM-CSF	CCATGATGGCCAGCCACTAC CTGGCTCCCAGCAGTCAAAG	141	60	20
MCP-1	GTCTCTGCCGCCCTTCTGTGC AACAGCAGGTGACTGGGGCA	100	60	10
PAI-1	CAGACCAAGAGCCTCTCC ATCACTTGGCCCATGAAAAG	202	54	20
vWF	GGCAATTCCTTCCTCCACAAAC CAGTTGACCCGATGACTCTTCA	167	61	20
TF	CACAGAGTGTGACCTCACCG ATTGTTGGCTGTCCGAGGTT	177	60	20
ICAM-1	CTGATGGGCAGTCAACAGCTA GCAGCGTAGGGTAAGGTTCT	116	60	10
VCAM-1	AGTCCCTGGAAACCAAGAGT TGCAGCTTTGTGGATGGATT	199	58	20
β-actin	GCACAGAGCCTCGCCTT CCTTGCACATGCCGGAG	112	61	20
GAPDH	CTCAACACGGGAAACCTCAC CGGACATCTAAGGGCATCAC	268	56	20

Cycling conditions were as follows: initial denaturation for 3 min at 95 °C, followed by 44 cycles of 10 s denaturation at 95 °C, 10 s annealing at X °C, and extension at 72 °C for Y s. Abbreviation: GAPDH = glyceraldehyde-3-phosphate dehydrogenase, GM-CSF = granulocyte-macrophage colony-stimulating factor, ICAM-1 = intercellular adhesion molecule 1, IL-6 = interleukin-6, IL-8 = interleukin-8, MCP-1 = monocyte chemoattractant protein 1, PAI-1 = plasminogen activator inhibitor-1, TF = tissue factor, VCAM-1 = vascular cell adhesion molecule 1, vWF = von Willebrand factor.
